# New Appliances and Things Medical

**Published:** 1897-09-25

**Authors:** 


### NEW APPLIANCES AND THINGS MEDICAL.

[We shall be glad to receive, at our Office, 28 & 29, Southampton Street, Strand, London, W.C., from the manufacturers, specimens of all new preparations and appliances which may be brought out from time to time.]

### BÉRAUDINE.

(Manufactured by the Peat Industries Syndicate, Limited, 15, Walbrook, E.C.)

The valuable properties of peat have long been known to the world, and practical use has been made of it in its natural state as a deodoriser and disinfectant, but it is only of recent years that attempts have been made to manufacture it into a substance which, while retaining all the wonderful qualities that first caught the attention of the public, should at the same time be convenient and acceptable to the medical profession for the dressing of wounds and other purposes. The great advantage this peat-fibre preparation, Béraudine, possesses, and which alone is almost sufficient to raise it to an eminent place among surgical dressings, is its power of deodorising; this speaks for itself. There is still a long list of inherent virtues to be noticed in regard to it; (1) its absorbing power is extraordinarily high; it is stated that it absorbs, gradually and continuously, liquid equal to fourteen times its own weight, and gases equal to at least nine times its own volume. (2) It is aseptic and antiseptic, undergoing no change in itself in any condition or circumstance, and effectually killing all microbes when brought into contact with them. (3) Absorbed liquids are readily diffused and evaporated by its agency. (4) It is so elastic and supple that it does not become matter and irritate the wound, but may remain for weeks on the wound and be perfectly comfortable to the patient and free from odour when withdrawn. It is very porous, absorbing the pus as quickly as it is formed, and from the acid which it contains causes the blood to coagulate. So thoroughly has its reputation been established that we hear that the French War Department has definitely adopted it instead of the gauzes and wool hitherto used in military hospitals. Peat flannel has all the advantages of wool and none of its defects, and is unique as a hygienic material, possessing as it does the properties of Béraudine. It is excellent, therefore, for travellers, cyclists, athletes, invalids, and, in fact, everybody.

### ALBENE.

Albene is a vegetable fat, daintily prepared and placed on the market by Messrs. Broomfield and Co., Upper Thames Street, E.C. Its appearance is greatly in its favour, being delicately white and smooth; and in this case appearances are not deceitful, for, besides being a good cooking agent, superior to animal fat in many ways, it also forms an excellent basis for ointment—much better than lard and vaseline. Vegetarians should welcome this new product, for it makes delicious pastry, and is in itself most digestible and nutritious. It has neither taste nor smell, and—great recommendation—does not turn rancid. Enough has been said, and it would seem to make every housekeeper eager to try Albene. It is sold in 1 lb., 2 lb., 3 lb., and 7 lb. tins, also in casks. Samples sent free to medical men.

